# Case Report: Fish bone-induced duodenal perforation leading to bile duct stones and portal vein thrombosis

**DOI:** 10.3389/fmed.2025.1546707

**Published:** 2025-04-01

**Authors:** Yuyi Shi, Xiliang Xu, Yuxu Wang, Wenhao Qu

**Affiliations:** Hepatobiliary and Pancreatic Medical Center, The First Affiliated Hospital of Shandong Second Medical University (Weifang People’s Hospital), Weifang, China

**Keywords:** duodenal foreign body, perforation, common bile duct stones, portal vein thrombosis, infection

## Abstract

We present a case of duodenal perforation caused by accidental ingestion of a fishbone, presenting with right upper abdominal pain and jaundice. Imaging studies revealed duodenal perforation, common bile duct stones, and portal vein thrombosis. By reviewing the patient’s clinical presentation, diagnostic process, treatment measures, and prognosis, we analyzed the potential mechanisms underlying this complex pathological condition and proposed appropriate clinical management strategies. Duodenal perforation caused by foreign bodies is a rare but potentially fatal condition that may lead to severe complications. Foreign body-induced duodenal and bile duct perforation can act as potential triggers for common bile duct stones and portal vein thrombosis. Its intricate pathophysiology requires heightened vigilance and comprehensive evaluation. Prompt diagnosis and individualized treatment strategies are critical for improving patient outcomes.

## Introduction

Duodenal perforation caused by foreign bodies is a rare but serious condition that may result in various complications, including biliary obstruction and portal vein thrombosis (PVT). As a precipitating factor for the formation of common bile duct stones and PVT, duodenal perforation represents a unique and complex clinical scenario requiring timely diagnosis and management.

Foreign body ingestion is a relatively common phenomenon, often presenting with asymptomatic or mild gastrointestinal symptoms. However, in rare cases, ingested foreign bodies may lead to severe gastrointestinal injuries such as perforation, especially in anatomically or pathologically weakened regions of the gastrointestinal tract. Perforation frequently triggers local or systemic inflammatory responses, which may extend to adjacent structures, including the biliary system and vascular networks (1).

The occurrence of common bile duct stones following duodenal perforation is an unusual phenomenon. Mechanical obstruction or subsequent scar formation caused by the foreign body may alter bile flow, creating a conducive environment for stone formation. Simultaneously, duodenal perforation may provoke adjacent vascular injury or infection, leading to a hypercoagulable state and predisposing the patient to complications such as PVT. Portal vein thrombosis is a significant clinical concern due to its potential to impair liver function and exacerbate systemic inflammatory responses (2).

While the isolated occurrences of common bile duct stones and PVT have been extensively reported, the simultaneous occurrence of both secondary to foreign body-induced duodenal perforation remains poorly understood. The literature on this topic is limited, with only a few case reports describing such presentations. These cases emphasize the importance of timely imaging studies and endoscopic interventions to prevent further complications (3).

In this report, we present a rare case of duodenal perforation caused by foreign body ingestion, which subsequently led to the development of common bile duct stones and PVT. Through this case, we aim to highlight the complex interplay between duodenal injury, biliary system complications, and vascular events. Additionally, we discuss the diagnostic challenges and therapeutic strategies involved in managing this intricate condition.

## Case description

A 60-year-old male patient was admitted to the gastroenterology department with “abdominal discomfort for 5 days.” Five days prior, the patient experienced abdominal discomfort without obvious cause, localized to the upper abdomen, unrelated to food intake, accompanied by back pain, fever (maximum temperature of 38.3°C), dark urine, and difficulty urinating. There was no nausea, vomiting, acid reflux, heartburn, or significant abdominal distension. At the emergency department, urinary catheterization alleviated the urinary symptoms. After self-medicating, the abdominal discomfort partially subsided. He presented to the outpatient clinic for further diagnosis and treatment. Laboratory tests revealed: ALT 102 U/L, AST 60 U/L, ALP 209 U/L, GGT 295 U/L, TBILI 54.2 μmol/L, D-BIL 35.0 μmol/L, WBC 10.00 × 10^9/L,CRP 21.1 mg/L. Abdominal ultrasound indicated liver cysts, intrahepatic hyperechoic areas, possible intrahepatic bile duct stones, dilation of the upper common bile duct, gallstones with inflammatory changes, and an accessory spleen. The patient was admitted for “liver dysfunction of unknown cause.”

**FIGURE 1:**
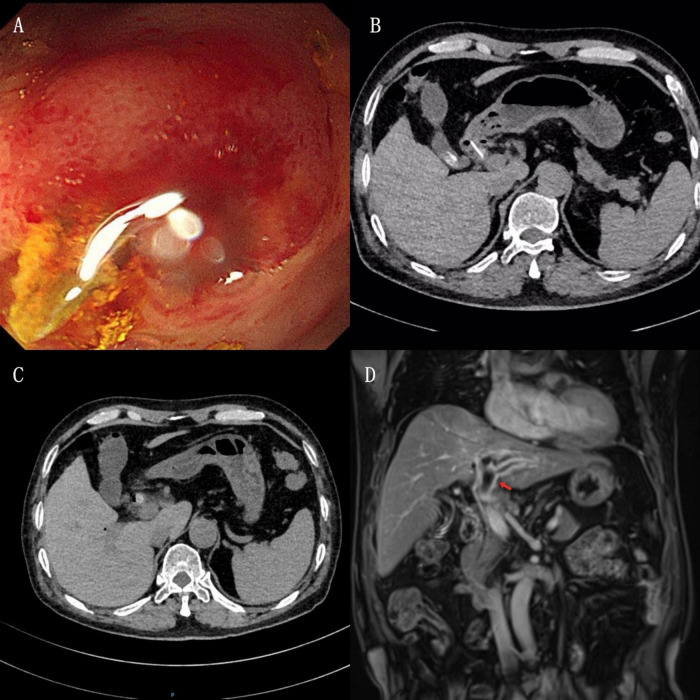
**(A)** Gastroscopy shows a sharp white foreign body in the duodenal bulb, partially embedded in the intestinal wall. **(B)** CT plain scan reveals a high-density shadow in the duodenum, closely associated with the intestinal wall. High-density shadows are visible in the gallbladder, consistent with gallstones. **(C)** CT plain scan shows a high-density shadow in the duodenum, slightly smaller than before. Common bile duct stones are observed, with a suspected duodenal foreign body extending into the common bile duct. **(D)** Contrast-enhanced MRI shows thrombus formation in the left branch of the portal vein (indicated by the red arrow).

Past medical history included 17 years of hypertension with a peak systolic pressure of 180 mmHg. The patient had an appendectomy over 20 years ago and bilateral inguinal hernia repair 9 months prior. The patient’s medical history is notable for more than 30 years of heavy alcohol use, with an average daily consumption of approximately 200 mL of 40% ABV spirits and frequent episodes of binge drinking. The patient also has a 30-year history of smoking, averaging 7 cigarettes per day, and has not attempted smoking cessation. During the hernia surgery, an upper abdominal CT scan identified a high-density area in the gastric antrum, suspected to be a foreign body, which was not addressed due to the absence of symptoms. Upon admission, MRI with dynamic contrast and MRCP indicated gallstones, intrahepatic and extrahepatic bile duct dilation, low signal in the bile ducts and gallbladder, biliary aerobilia, and portal vein thrombus formation. Abdominal CT revealed common bile duct stones and a suspected foreign body extending from the stomach into the common bile duct (Figure 1). Upon further inquiry into the patient’s history, the patient reported that 9 months ago during inguinal hernia surgery (the patient has a history of smoking and was hospitalized for interstitial pneumonia 2 years before the surgery), a chest CT scan revealed a foreign body in the gastric antrum. A subsequent gastroscopy indicated the presence of a foreign body in the duodenal bulb, and surgical management was recommended; however, the patient and family refused. After consultation with our department, the patient was transferred for further treatment. Preoperatively, the patient received piperacillin-tazobactam for anti-infective therapy, and surgical treatment was performed on January 11, 2024.

## Surgical procedure

**FIGURE 2:**
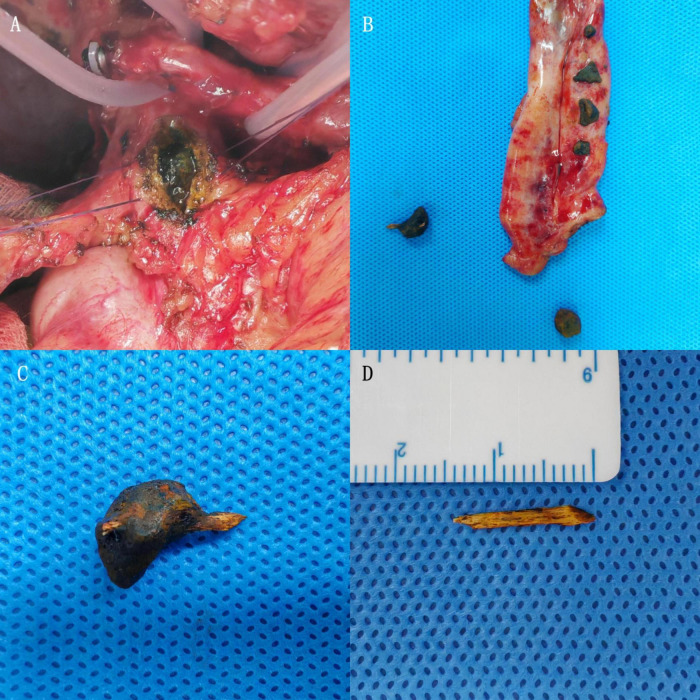
**(A)** Multiple stones observed in the common bile duct during surgery. **(B)** After gallbladder removal and dissection, multiple stones are visible inside the gallbladder and common bile duct. **(C)** One larger common bile duct stone contains a white, high-density foreign body traversing it, suspected to be a fishbone. **(D)** After removing the stone’s surface, a hard, white foreign body is revealed, approximately 1.4 cm in length, with sharp ends.

Under general anesthesia, the patient underwent common bile duct exploration with stone extraction, T-tube drainage, cholecystectomy, portal vein thrombectomy, duodenotomy with foreign body removal, intraoperative cholangioscopy, adhesiolysis, and laparoscopy. The patient first underwent laparoscopic surgery, Laparoscopic exploration revealed nodular liver cirrhosis, partial abdominal adhesions, and dense adhesions between the gallbladder, mesentery, stomach, and duodenum, necessitating conversion to open surgery. Extensive upper abdominal adhesions were lysed, exposing the liver and hepatoduodenal ligament. The common bile duct was densely adhered to the duodenal bulb, with the gallbladder congested and edematous. Upon incising the common bile duct and duodenal bulb, three stones were identified, one of which encased a 1.4 cm elongated white fishbone-like foreign body, partly extending into the duodenum (Figure 2). The duodenal and bile duct perforation with an internal fistula and chronic inflammation was repaired with absorbable sutures. Intraoperative cholangioscopy confirmed no residual stones in the bile ducts.

A 16F T-tube was placed, and the bile duct was sutured with absorbable material. Dissection around the portal vein revealed a hardened left branch without detectable blood flow on ultrasound. The main portal vein trunk was clamped, and the left branch was partially incised, revealing a molded thrombus with a firm, organized structure that was challenging to extract. Partial thrombus removal was sent for frozen pathological examination, which showed proliferative fibrous and smooth muscle tissue with hemorrhage and inflammatory infiltration, without evidence of malignancy. The portal vein was flushed with heparinized saline and repaired with absorbable sutures. No blood leakage was observed upon releasing the vascular clamp. Hemostasis was achieved, and hemostatic gauze was applied. A single abdominal drainage tube and the T-tube were brought out through the abdominal wall. The patient’s postoperative recovery was stable and uneventful. He was discharged on postoperative day 12. At the 6-month postoperative follow-up, no recurrence of stones or complications was observed.

## Discussion

Regarding the mechanism of fishbone migration, we hypothesize that a relatively long fishbone, after entering the duodenum from the stomach and bending, is easily trapped in the folds of the duodenum by intestinal peristalsis. As the duodenum is retroperitoneal and flattened near the minor curvature on the papillary side, in contrast to the major curvature, a fishbone moving diagonally along the duodenal axis may inadvertently lodge into the duodenal folds. With continued peristalsis and subsequent food impact, the fishbone becomes embedded in the duodenal wall, eventually penetrating the wall and entering the bile duct (4). Previous studies have shown that food foreign bodies in the common bile duct may serve as a nidus for stone formation, leading to impaired bile drainage (5, 6). In our case, the stone formed around the fishbone was primarily composed of brown bilirubin. Bilirubin, in the presence of bacterial infections associated with bile stasis, binds to calcium salts with low solubility, leading to stone formation around the fishbone (7).

Foreign bodies most commonly found in the bile duct include surgical clips, stent fragments, and T-tube remnants. Foreign bodies that traverse the gastrointestinal tract into the bile duct include bezoars, fishbones, metal needles, chicken bones, and toothpicks. Accidental fishbone ingestion, particularly when asymptomatic, often goes unnoticed by patients, making clinical and radiological diagnosis challenging. Symptoms may appear months or even years later, complicating the diagnostic process (8). The patient in our case lived near a river and frequently consumed fish but had no recollection of recent fish consumption causing discomfort. The fishbone discovered during a prior inguinal hernia surgery was incidental. Despite the attending physician’s recommendation for surgical treatment, the patient and family refused, opting for observation due to the absence of significant symptoms.

For isolated foreign body-induced stones, minimally invasive treatment with endoscopic sphincterotomy for stone removal is often recommended. There have been reports of successful retrieval of fishbones post-pancreaticoduodenectomy via endoscopy (9). However, for patients with dual perforations and severe infections, surgical treatment is often necessary (10). Our patient presented with cholecystolithiasis, cholecystitis, pneumobilia, and suspected portal vein thrombus, potentially caused by recurrent biliary infections. Distinguishing between a thrombus and tumor thrombus was challenging. After multidisciplinary discussion, surgery was deemed the optimal treatment. This approach allowed exploration and repair of a potential duodenal-common bile duct fistula and identification of the portal vein thrombus, which was later confirmed via intraoperative pathology as a thrombus.

It is well established that the causes of portal vein thrombosis (PVT) include chronic liver disease (cirrhosis), primary and secondary hepatobiliary malignancies, major infectious or inflammatory abdominal conditions, and myeloproliferative disorders (11). In this patient, intraoperative findings revealed a relatively firm liver texture and a cirrhotic appearance, making cirrhosis-related PVT a possibility. Taking into account the patient’s long history of heavy alcohol use, normal virological serology results, and intraoperative findings suggestive of altered liver tissue, we surmise that the cirrhosis is most likely attributable to alcoholic liver disease.

However, previous imaging studies did not identify any portal vein thrombus, and intraoperatively, marked inflammation, adhesions, and thickening of the portal vein wall were noted only in the vicinity of the fish bone–associated perforation. The remaining segments of the portal vein appeared unremarkable, and the patient showed no overt signs of portal hypertension—such as splenomegaly, esophagogastric varices, or thrombocytopenia. Therefore, we are inclined to believe that the immediate precipitating factor for this episode of portal vein thrombosis is more closely related to the perforation and localized infection caused by the fish bone.

Portal vein thrombosis caused by biliary infections is rare, with few cases reported in the literature. These include massive abdominal hemorrhage due to portal vein thrombosis caused by acute cholangitis and cholecystolithiasis, acute portal vein obstruction secondary to cholangitis post-Kasai procedure for biliary atresia, and portal vein thrombosis following liver transplantation complicated by chronic cholangitis (12, 13). The pathogenesis of portal vein thrombosis involves endothelial injury, blood stasis, and hypercoagulability. Acute cholangitis-associated portal vein thrombosis is thought to result from direct inflammation spreading to the portal vein, causing endothelial injury and hypercoagulable states (13).

In this case, the intraoperative thrombus in the portal vein was mold-shaped, firm, and organized, tightly adherent to the portal vein wall, making separation and removal challenging. We considered it a result of recurrent cholangitis causing endothelial injury combined with hypercoagulability, leading to portal vein thrombosis. The thrombus appeared chronic and primarily affected the left branch of the portal vein, with minimal impact on hepatic function and limited benefit from revascularization. Thus, further intervention was abandoned intraoperatively.

This study is a single-case report and lacks a more comprehensive evaluation of the staging of liver cirrhosis (such as liver biopsy or liver elastography) as well as experimental verification of the mechanism of portal vein thrombosis formation. Additionally, after discharge, the patient only underwent one follow-up at our hospital at 6 months postoperatively, and no further follow-ups were conducted, which limits our understanding of his long-term liver function and the status of the portal vein thrombosis.

Duodenal perforation caused by fishbone ingestion is relatively rare, and the occurrence of perforation involving the common bile duct is particularly uncommon. We report a case in which fishbone ingestion led to perforations of both the duodenum and the bile duct, resulting in the formation of a common bile duct stone and portal vein thrombosis, which were successfully addressed through surgery. We hope this case report provides valuable reference material for the diagnosis and treatment of similar conditions. Furthermore, it serves as a typical example of multidisciplinary management for surgeons, gastroenterologists, and radiologists. Through the discussion of this case, we aim to offer some insights into the diagnosis and treatment approaches for complications arising from perforation or foreign body ingestion encountered in clinical practice.

## Data Availability

The original contributions presented in this study are included in this article/supplementary material, further inquiries can be directed to the corresponding author.
